# Supplementary material for: Large-scale genome-wide association studies reveal the genetic causal etiology between air pollutants and autoimmune diseases
Source: J Transl Med. 2024 Apr 29;22:392. doi: 10.1186/s12967-024-04928-y (PMC11057084; doi:10.1186/s12967-024-04928-y)
Supplement: Supplementary file 1 — Additional file 1: Supplementary methods and figures. Figure S1. The leave-out-one plot for air pollution and autoimmune diseases. Figure S2. TSMR meta-analysis between air pollution and autoimmune diseases. Figure S3. Hub gene transcripts analysis associated with air pollution and autoimmune diseases. [file 12967_2024_4928_MOESM1_ESM.docx]

Supplementary Information

Supplementary Figures


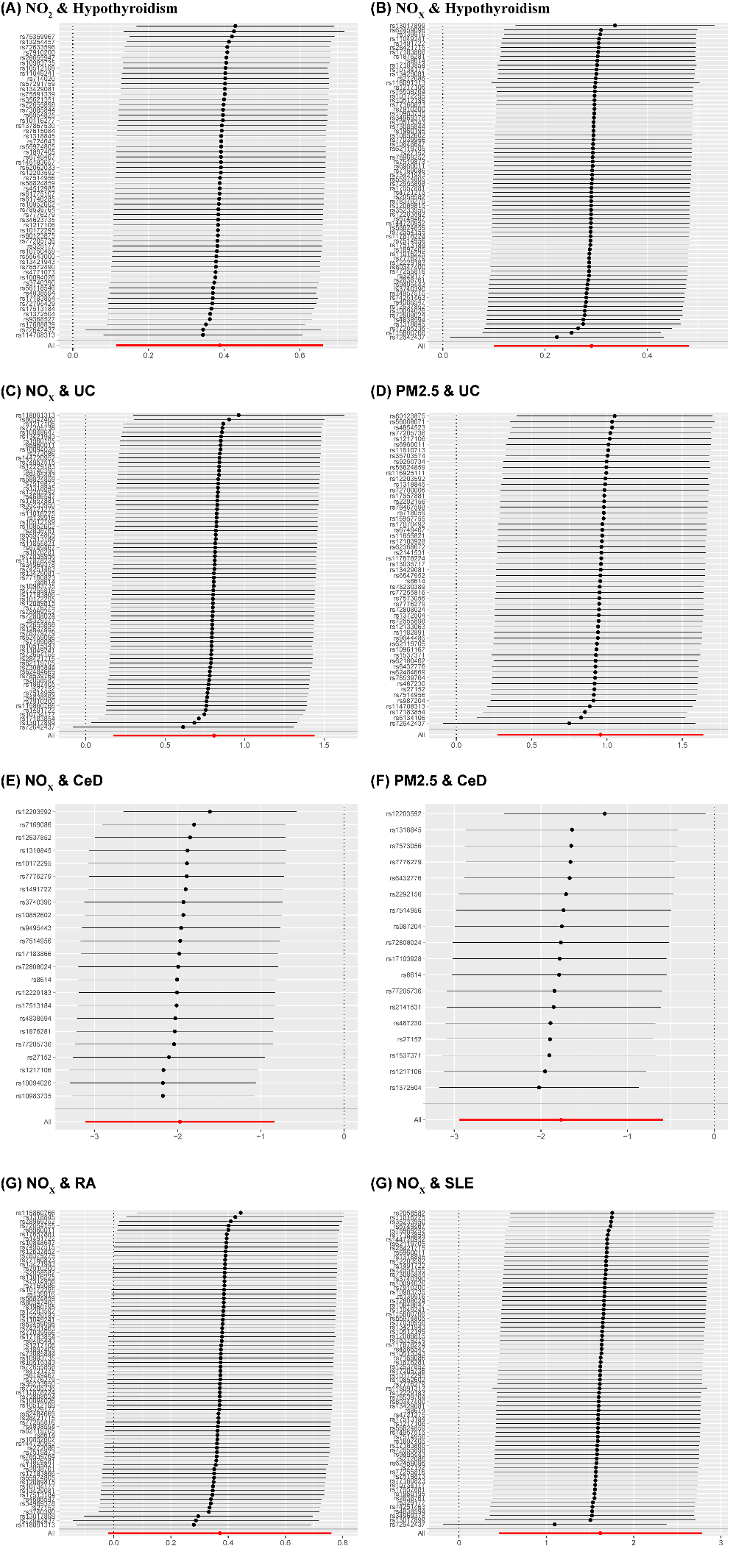


Supplementary Figure 1. The leave-out-one plot for air pollution and autoimmune diseases.


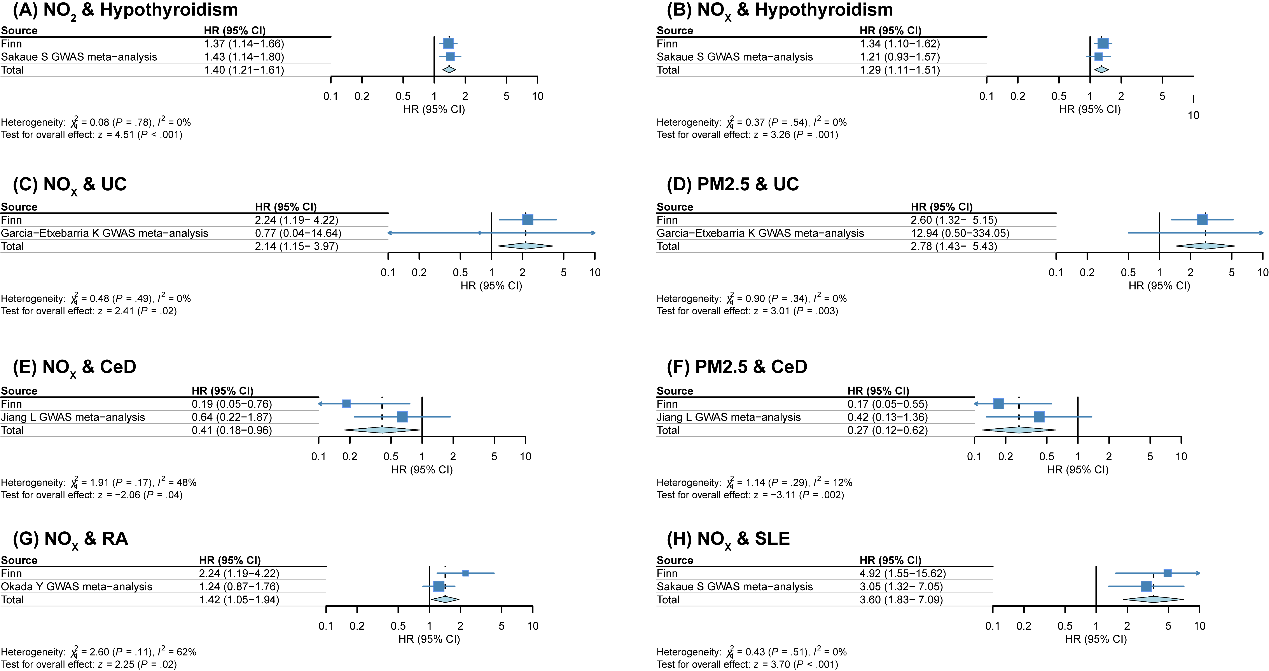


Supplementary Figure 2. TSMR meta-analysis between air pollution and autoimmune diseases.


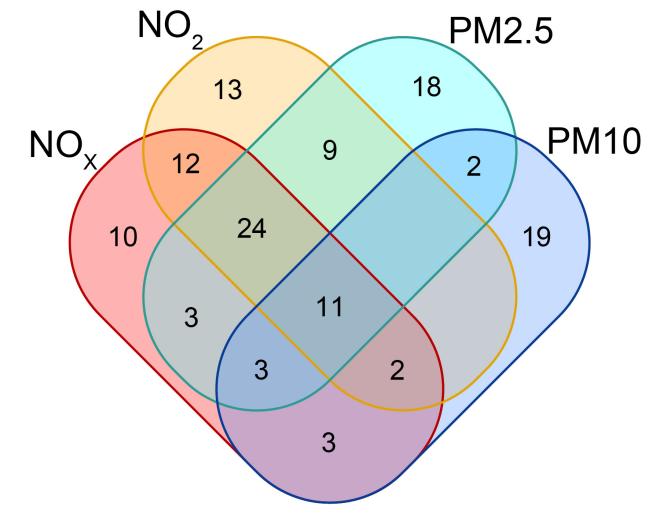


Supplementary Figure 3. Hub gene transcripts analysis associated with air pollution and autoimmune diseases.

Supplementary Methods

Detailed sources and descriptions of data

Air pollution

The summary genetic association statistics for particulate matter air pollution 10 (PM10) (GWAS ID: ukb-b-18469) and particulate matter air pollution 2.5 (PM2.5) (GWAS ID: ukb-b-10817) were obtained from a recent residential air pollution GWAS published in 2018 based on 423,796 individuals and 9,851,867 SNPs of European ancestry from the UK Biobank (UKB) prospective cohort study (https://biobank.ndph.ox.ac.uk/ukb). The summary genetic association statistics for nitrogen oxides (NO_X_) (GWAS ID: ukb-b-12417) and nitrogen dioxide (NO_2_) (GWAS ID: ukb-b-9942) were obtained from a recent residential air pollution GWAS published in 2018 based on 456,380 individuals and 9,851,867 SNPs of European ancestry from the UK Biobank (UKB) prospective cohort study. All air pollutants were measured in micrograms/m^3^. In the year 2010, approximations of air pollution were evaluated via Land Use Regression (LUR) utilizing a model that was devised as part of the European Study of Cohorts for Air Pollution Effects (ESCAPE) (http://www.escapeproject.eu/) and was funded under the EU 7th Framework Programme. ESCAPE conducted monitoring from 26 Jan 2010 - 18 January 2011, thereby enabling air pollution evaluations that reproduce 2010's conditions. Significantly, traffic variables were calculated with the aid of a geographic information system (GIS) during the LUR's progression. The maximum value of PM10 was 31.39 micro-g/m^3^, the minimum value was 11.78 micro-g/m^3^, and the average value was 16.24 micro-g/m^3^; the maximum value of PM2.5 was 21.31 micro-g/m^3^, the minimum value was 8.17 micro-g/m^3^, and the average value was 9.99 micro-g/m^3^; the maximum value of NO was 265.94 micro-g/m^3^, the minimum value was 19.74 micro-g/m^3^, and the average value was 44.11micro-g/m^3^; the maximum value of NO^2^ is 108.49 micro -g/m^3^, the minimum value was 12.93 micro-g/m^3^, and the average value was 26.71 micro-g/m^3^.

The UK Biobank represents a vast biomedical archive and scholarly information source featuring comprehensive health and genetic data that has been submitted by roughly five hundred thousand United Kingdom-based participants [1]. It routinely encompasses supplementary data sets, and researchers who are authorized can access it internationally for important research on the most widespread and life-threatening illnesses. It provides a significant contribution to modern medicine and therapy and has facilitated many essential scientific findings that improve health outcomes. UKB encompasses biological samples and data for a multitude of people totaling around five hundred thousand, who were recruited between 2006 and 2011 via twenty-two UKB assessment centers located in England, Wales, and Scotland. The participants, aged between 40 and 69 years, provided baseline data that included questionnaires, physical and cognitive tests, and blood and urine samples. At the moment of participation, males and females had an estimated mean age of 56.63 years and 57.10 years, respectively.

Potential confounders

The summary genetic association statistics for body mass index (BMI) were obtained from an obesity biology GWAS meta-analysis published in 2015 based on 339,224 individuals from 125 studies, 82 with GWAS results (n = 236,231) and 43 with results from Metabochip (n = 103,047) [2]. This GWAS contains 104,666 European-ancestry male individuals, 132,115 European-ancestry female individuals, 370 African American male individuals, 517 African American female individuals, 512 Hispanic male individuals, and 764 Hispanic female individuals. Subsequent to carrying out regression analysis on age and gender, as well as the inverse normal transformation of residuals, the authors executed association analyses utilizing genotypes or imputed genotype dosages. To create a GWAS, they amalgamated all relevant data, as per Metabochip studies, and followed this with a GWAS plus Metabochip combined meta-analysis. Their sample consisted of 322,154 individuals of European descent, in addition to 17,072 individuals of non-European descent, whose data we thoroughly analyzed. In this paper, we extracted the GWAS data of BMI for the European population only, with a total of 236,781 individuals and 2,529,499 SNPs (GWAS ID: ebi-a-GCST002783).

The summary genetic association statistics for smoking were obtained from the Within Family GWAS consortium published in 2022 [3]. The estimates emanating from genome-wide association studies (GWAS) of unrelated individuals encompass a variety of effects, such as those of inherited variation (direct effects), demography (population stratification, assortative mating), and relatives (indirect genetic effects). Estimates for within-family genetic association, as obtained from sibling samples, tend to be less biased in terms of direct genetic effects since they are less likely to be influenced by parent-derived demographic and indirect genetic effects. The extent of within-sibship GWAS has hitherto been limited by the available data but now combines well-established family studies with the recent, large biobanks that happen to or are designed to hold thousands of sibships, making it quite feasible. As such, the researchers combined data from 19 cohorts made up of 178,086 siblings (with sample sizes ranging from 13,375 to 163,748, including European, Asian, and American populations) to produce population (between-family) and within-sibship estimates for 25 phenotypes. It was observed that within-sibship GWAS estimates were smaller compared to population estimates for height, educational attainment, age at first birth, number of children, cognitive ability, depressive symptoms, and smoking. In this paper, we extracted the GWAS data of ever smoked for the European population only, with a total of 99,996 individuals and 7,933,821 SNPs (GWAS ID: ieu-b-4858).

The summary genetic association statistics for alcoholic drinks per week were obtained from the GWAS and Sequencing Consortium of Alcohol and Nicotine use consortium published in 2019 [4]. In a sample size of up to 1.2 million Europeans (52.2% female), the authors found 566 genetic variants in 406 loci associated with multiple stages of tobacco use (initiation, cessation, and heavy) and alcohol use, with 150 loci showing polymorphic associations. The selection of phenotypes was carried out based on their availability in a substantial number of participating studies, previous genetic association research outcomes for each phenotype, and their clinical significance. Following extensive consultations and the gathering of pertinent information, phenotypes were chosen and defined. All studies were directed to employ age, age squared, sex, and genetic principal components as covariates in the genetic association analysis. Similarly, they were asked to conduct a correction of cigarettes per day with regard to the current and prior smoker status (if available). Additionally, all studies were requested to consider the employment of other study-specific covariates in their analyses (e.g., site in multi-site studies, case-control status). The variable 'alcohol use' was defined as the mean number of drinks reported by each participant for consumption per week, covering all types of alcohol. In instances where a study had recorded response ranges in bins (e.g. 1-4 drinks per week, 5-10 drinks per week), we employed the midpoint of the given range. For example, if an individual stated 1-5 drinks per week, his average weekly consumption was deemed 2.5 drinks per week. A variety of measures were used, such as "Thinking about the past year, on average, how many drinks did you have each week?" or "In the past week, how many alcoholic beverages did you have?". This phenotype was left-anchored at one and log-transformed prior to analysis to prevent any outliers from exerting undue influence on the analyses. In this paper, we extracted the GWAS data of alcohol use for the European population only, with a total of 335,394 individuals and 11,887,865 SNPs (GWAS ID: ieu-b-73).

The summary genetic association statistics for income were obtained from a recent household income before taxes GWAS published in 2018 based on 397.751 individuals and 9,851,867 SNPs of European ancestry from the UKB prospective cohort study (GWAS ID: ukb-b-7408). Participants receive the touch screen question, "What is your average gross household income before taxes?" If participants activate the "Help" button, they are presented with this information: If unsure of the annual household income, there are the weekly and monthly equivalents. There are 7 results in total: less than £18,000, £18,000 to £30,999, £31,000 to £51,999, £52,000 to £100,000, more than £100,000, don't know, and prefer not to answer. The instrumental variants of income extracted from this GWAS represented the relative income level among participants[5].

Autoimmune diseases

The summary genetic association statistics for T1D were obtained from a recent residential T1D GWAS published in 2021 [6]. The authors performed deep imputation of genotyping data followed by genome-wide association testing and meta-analysis on 9,358 type 1 diabetes cases and 15,705 control subjects from 12 European cohorts. Candidate variants were replicated in separate cohorts of 4,329 cases and 9,543 control subjects. In this paper, we extracted the GWAS data of T1D for the European population only, with a total of 24,840 individuals and 12,783,129 SNPs (GWAS ID: ebi-a-GCST010681).

The summary genetic association statistics for CD were obtained from the International Inflammatory Bowel Disease Genetics Consortium published in 2016[7]. After quality control and imputation of 1000 genomes, 5,956 Crohn's disease cases, 6,968 ulcerative colitis cases, and 21,770 population controls of European ancestry were used for genome-wide association studies of Crohn's disease, using an additional 16,619 Crohn's disease cases, 13,449 ulcerative colitis cases, and 31,766 population controls in Immunochip replication was performed. The replication cohort consisted of 2,025 Crohn's disease cases, 2,770 ulcerative colitis cases, and 5,051 population controls of non-European ancestry, and thus individuals were assigned to one of four ancestral groups (European, Iranian, Indian, or East Asian) using principal component analysis. In this paper, we extracted the GWAS data of CD for the European population only, with a total of 20,883 individuals and 12,276,506 SNPs (GWAS ID: ieu-a-30).

The summary genetic association statistics for CeD were obtained from a recent celiac disease influencing immune gene expression GWAS published in 2021 [8]. The authors performed a second-generation genome-wide association study of 4,533 celiac disease cases and 10,750 controls, yielding a total of 518,292 SNPs (GWAS ID: ebi-a-GCST000612).

The summary genetic association statistics for MS were obtained from the International Multiple Sclerosis Genetics Consortium published in 2019 [9]. The authors analyzed genetic data of 47,429 MS and 68,374 control subjects and established a reference map of the genetic architecture of MS that includes 200 autosomal susceptibility variants outside the major histocompatibility complex, one chromosome X variant, and 32 variants within the extended MHC, and yielding a total of 6,304,359 SNPs (GWAS ID: ieu-b-18).

The summary genetic association statistics for PSC were obtained from a recent PSC GWAS published in 2017 [10]. The author undertook the largest genome-wide association study of PSC (4,796 cases and 19,955 population controls) and identified four new genome-wide significant loci. In this paper, we extracted the GWAS data of PSC for the European population only, with a total of 14,890 individuals and 7,891,603 SNPs (GWAS ID: ieu-a-11121).

GWAS data for other autoimmune diseases were obtained from the FINNGEN consortium published on May 11, 2021, with a total of 218,792 sample sizes and 16,962,023 variants (https://www.finngen.fi/en/access_results). FINNGEN constitutes one of the few personalized medicine ventures conducted on such a scale, with collaborative efforts made between public and private entities EN MASSE, resulting in a uniquely exceptional and unparalleled project when compared to several other ongoing studies. The study, which was launched in Finland during the fall of 2017, presents a distinctive endeavor integrating genome information with digital healthcare data. This unparalleled study is considered to be one of the largest of its kind, with the intention of improving human health through genetic research by identifying novel therapeutic targets and diagnostics to treat a myriad of illnesses. Remarkably, the collaborative nature of this project is unique when compared to other ongoing studies, with all the partners working together tightly to ensure transparency, data security, and ownership concerns are thoroughly considered. FINNGEN unifies Finnish universities, hospital districts, and biobanks, as well as international pharmaceutical corporations and, potentially, hundreds of thousands of Finns themselves. Indeed, cooperation among all members is the critical component toward achieving breakthroughs in disease prevention, diagnosis, and treatment.

Selection for Instrumental variables

This paragraph outlines the method used to select genetic instruments in a multivariable Mendelian randomization (MVMR) analysis. The aim is to identify genetic variants that can be used as instrumental variables to estimate the causal effect of an exposure on an outcome. To select the genetic instruments, we first set a genome-wide *p*-value threshold of 5e-6 for correlations between the genetic variants and the exposures to air pollution. This is a stringent threshold designed only to allow the most robust associations to be included in the analysis. Next, we conducted a linkage disequilibrium analysis to identify genetic variants that were not in high linkage disequilibrium with each other (r^2^ < 0.001 and distance < 10MB). This was done to ensure that the selected instruments were not tagging the same underlying genetic variation. We then removed any SNPs that were shared between the exposures and the outcomes, as these could introduce bias into the analysis.

To further refine the selection of instruments, we calculated the F statistics for each instrument in the exposures. The F statistic is a measure of the strength of the instrumental variable and is calculated based on the number of genetic variants (K), the sample size (N), and the variance explained by the genetic variants (R^2^). Instruments with an F statistic of less than ten were excluded, as they were deemed insufficiently robust to provide reliable estimates of the exposure-outcome relationship.

Overall, this process of selecting genetic instruments ensures that only the most robust and independent genetic variants are included in the MVMR analysis, thereby increasing the validity of the causal inferences drawn from the results.

Two-sample Mendelian randomization (TSMR)

This paragraph describes the statistical methods used to conduct TSMR (two-sample Mendelian randomization), a type of MR analysis that uses summary-level data from different sources to estimate causal effects. Three different methods were used to conduct the TSMR analysis: random-effect inverse-variance weighted (IVW), weighted median, and MR Egger. IVW was used as the main result method and was calculated by taking the weighted regression of the SNP-outcome effects and SNP-exposure effects with the intercept constrained to zero. This method assumes that all instruments are valid and that there is no pleiotropy (when one genetic variant affects multiple traits) [11]. Weighted median and MR Egger were used as supplementary results methods because they provide more robust estimates in broader conditions[12, 13]. However, they are less efficient than the IVW method.

Multivariate Mendelian randomization (MVMR)

This paragraph describes the use of MVMR (multivariable Mendelian randomization) to estimate the multiple effects or exposures on an outcome and how it was used to estimate the more direct effects of air pollution on autoimmune diseases. MVMR is a variant of MR analysis that allows the estimation of the effects of multiple exposures or confounding factors on an outcome[14]. These exposures can include confounders, mediators, or colliders, which can make the estimation of direct effects more challenging[15]. MVMR is also suitable for accounting for pleiotropic variants.

In this study, MVMR was used to estimate the direct effects of air pollution on autoimmune diseases. The analysis adjusted for several potential confounding variables, including BMI, alcohol intake frequency, number of cigarettes previously smoked daily, and income. By accounting for these variables, MVMR allowed for a more accurate estimation of the direct effect of air pollution on autoimmune diseases while controlling for potential confounding factors.

Sensitivity Analyses

To appraise the heterogeneity in genetic instruments' effects to identify potential violations of the instrumental variable (IV) assumptions, we administered the MR Egger intercept test [13], the Cochran Q heterogeneity test[12], as well as the MR pleiotropy residual sum test[16]. MR-Egger regression intercept is fundamentally interpreted as the mean pleiotropic effect across all instruments: thus, MR-Egger regression confers a test for average pleiotropy and has been extended to correct for both measured and unmeasured pleiotropy in TSMR. In MR, the Cochran Q test, typically employed to identify outliers in regression analysis, denotes average pleiotropy, which can induce heterogeneity in individual ratio estimates.

Transcriptome-wide association study (TWAS) and enrichment of biological pathways

This paragraph describes the methodology used for transcriptomic imputation and subsequent analysis of gene expression to identify potential biological mechanisms underlying the relationship between air pollution and autoimmune diseases.

To conduct transcriptomic imputation, the GWAS data was converted into TWAS using the FUSION method [17]. FUSION uses an eQTL-based linear model to predict gene expression based on reference panels of RNA-seq data. In this study, European whole blood samples from GTEx v8 (N = 558) [18], the CommonMind Consortium’s (CMC) (N=452), and splicing (N = 452) [19] references were utilized as reference panels.

The TWAS results were analyzed to identify genes significantly associated with both air pollution and autoimmune disease in the same direction. To further understand the potential biological mechanisms underlying this relationship, biological pathway enrichment analyses were conducted for these genes. The Gene Ontology database was used in these analyses, and R packages of cluster Profiler, enrichplot, and DOSE were utilized for the analyses. By identifying key biological pathways associated with air pollution and autoimmune disease, the study aimed to shed light on the underlying mechanisms of this relationship.

Two-step Mendelian randomization

Mediation analysis intends to explain the pathway(s) through which an exposure afects an outcome. To investigate the potential mediators from air pollutants to autoimmune diseases with potential causality, two-step MR was performed. Summarized GWAS for the plasma proteome was collected from 35,559 Icelanders which included 4907 protein information[20]. We separate protein quantitative trait loci (pQTL) into cis-pQTL (within ±1 MB window of the gene encoding the corresponding protein) and trans-pQTL (outside ±1 MB window).

In the first step of two-step MR, proteome-wide MR was performed by using cis-pQTL (P<1×10^-5^, clump_kb=10000 and r^2^ = 0.001) (as exposure) to identify the potential proteins causally associated with the risk of autoimmune diseases (as outcome). The Proteins with FDR < 0.05 were deemed as risk proteins for autoimmune disease. In the second step, the effects of air pollutants (as exposure) on these risk proteins (as outcome) were assessed by MR. The mediating effect of risk proteins were calculated by beta1 (effects of pQTL on autoimmune diseases) * beta2 (effects of air pollutants on pQTL). Standard errors were estimated by delta method[21].

Colocalization analysis

Although LD clumping could filter strong and independent IVs for MR, the causal estimates could also be affected and by LD[22]. The genetic variants could be in LD with another variant which independently affects the outcome. In this case, the IVs for MR is invalid. To solve this issue, colocalization analyses between pQTLs and autoimmune diseases were conducted, which could powerfully detect the shared causal variants in a region between two traits[22]. Extracting the effect estimates and standard errors from summarized GWAS, the approximate Bayes factor for each SNP was calculated. Then log Bayes factor for each hypothesis was estimated[23]. An arbitrary posterior probability > 0.8 was set for each 4 hypotheses: PP.H0 – no causal variant; PP.H1 and PP.H2 – causal variant for one of the traits; PP.H3 – distinct causal variant for each trait; PP.H4 – shared causal variant for both traits. Default values was set for prior probabilities: P1 (SNP is associated with the pQTL) and P2 (SNV is associated with autoimmune diseases) were both set at 1e-4, and P12 (SNV is associated with both pQTL and the autoimmune diseases) was set at 1e-5[23]. IVs for cis-pQTL were identified as lead SNPs. All SNPs within 1 MB around the lead SNPs for pQTL and autoimmune diseases GWAS were extracted and calculated the posterior for H4 (PPH4, the probability of shared causal variant for both trait). A locus was deemed as colocalized if PPH4 > 0.8. The colocalized signals between pQTLs and autoimmune diseases could reinforce the causal effects between protein levels and autoimmune diseases.

Reference

1. Bycroft C, Freeman C, Petkova D, Band G, Elliott LT, Sharp K, Motyer A, Vukcevic D, Delaneau O, O'Connell J *et al*: **The UK Biobank resource with deep phenotyping and genomic data**. *Nature* 2018, **562**(7726):203-209.

2. Locke AE, Kahali B, Berndt SI, Justice AE, Pers TH, Day FR, Powell C, Vedantam S, Buchkovich ML, Yang J *et al*: **Genetic studies of body mass index yield new insights for obesity biology**. *Nature* 2015, **518**(7538):197-206.

3. Howe LJ, Nivard MG, Morris TT, Hansen AF, Rasheed H, Cho Y, Chittoor G, Ahlskog R, Lind PA, Palviainen T *et al*: **Within-sibship genome-wide association analyses decrease bias in estimates of direct genetic effects**. *Nat Genet* 2022, **54**(5):581-592.

4. Liu M, Jiang Y, Wedow R, Li Y, Brazel DM, Chen F, Datta G, Davila-Velderrain J, McGuire D, Tian C *et al*: **Association studies of up to 1.2 million individuals yield new insights into the genetic etiology of tobacco and alcohol use**. *Nat Genet* 2019, **51**(2):237-244.

5. Ye C-J, Kong L-J, Wang Y-Y, Dou C, Zheng J, Xu M, Xu Y, Li M, Zhao Z-Y, Lu J-L *et al*: **Mendelian randomization evidence for the causal effects of socio-economic inequality on human longevity among Europeans**. *Nat Hum Behav* 2023, **7**(8):1357-1370.

6. Forgetta V, Manousaki D, Istomine R, Ross S, Tessier MC, Marchand L, Li M, Qu HQ, Bradfield JP, Grant SFA *et al*: **Rare Genetic Variants of Large Effect Influence Risk of Type 1 Diabetes**. *Diabetes* 2020, **69**(4):784-795.

7. Liu JZ, van Sommeren S, Huang H, Ng SC, Alberts R, Takahashi A, Ripke S, Lee JC, Jostins L, Shah T *et al*: Association analyses identify 38 susceptibility loci for inflammatory bowel disease and highlight shared genetic risk across populations. *Nat Genet* 2015, 47(9):979-986.

8. Dubois PC, Trynka G, Franke L, Hunt KA, Romanos J, Curtotti A, Zhernakova A, Heap GA, Adany R, Aromaa A *et al*: **Multiple common variants for celiac disease influencing immune gene expression**. *Nat Genet* 2010, **42**(4):295-302.

9. International Multiple Sclerosis Genetics C: Multiple sclerosis genomic map implicates peripheral immune cells and microglia in susceptibility. *Science* 2019, 365(6460).

10. Ji SG, Juran BD, Mucha S, Folseraas T, Jostins L, Melum E, Kumasaka N, Atkinson EJ, Schlicht EM, Liu JZ *et al*: Genome-wide association study of primary sclerosing cholangitis identifies new risk loci and quantifies the genetic relationship with inflammatory bowel disease. *Nat Genet* 2017, 49(2):269-273.

11. Burgess S, Butterworth A, Thompson SG: Mendelian randomization analysis with multiple genetic variants using summarized data. *Genet Epidemiol* 2013, 37(7):658-665.

12. Bowden J, Del Greco MF, Minelli C, Zhao Q, Lawlor DA, Sheehan NA, Thompson J, Davey Smith G: **Improving the accuracy of two-sample summary-data Mendelian randomization: moving beyond the NOME assumption**. *Int J Epidemiol* 2019, **48**(3):728-742.

13. Bowden J, Del Greco MF, Minelli C, Davey Smith G, Sheehan N, Thompson J: **A framework for the investigation of pleiotropy in two-sample summary data Mendelian randomization**. *Stat Med* 2017, **36**(11):1783-1802.

14. Sanderson E, Davey Smith G, Windmeijer F, Bowden J: An examination of multivariable Mendelian randomization in the single-sample and two-sample summary data settings. *Int J Epidemiol* 2019, 48(3):713-727.

15. Carter AR, Sanderson E, Hammerton G, Richmond RC, Davey Smith G, Heron J, Taylor AE, Davies NM, Howe LD: **Mendelian randomisation for mediation analysis: current methods and challenges for implementation**. *Eur J Epidemiol* 2021, **36**(5):465-478.

16. Verbanck M, Chen CY, Neale B, Do R: Detection of widespread horizontal pleiotropy in causal relationships inferred from Mendelian randomization between complex traits and diseases. *Nat Genet* 2018, 50(5):693-698.

17. Gusev A, Ko A, Shi H, Bhatia G, Chung W, Penninx BW, Jansen R, de Geus EJ, Boomsma DI, Wright FA *et al*: **Integrative approaches for large-scale transcriptome-wide association studies**. *Nat Genet* 2016, **48**(3):245-252.

18. Consortium GT: The GTEx Consortium atlas of genetic regulatory effects across human tissues. *Science* 2020, 369(6509):1318-1330.

19. Fromer M, Roussos P, Sieberts SK, Johnson JS, Kavanagh DH, Perumal TM, Ruderfer DM, Oh EC, Topol A, Shah HR *et al*: **Gene expression elucidates functional impact of polygenic risk for schizophrenia**. *Nat Neurosci* 2016, **19**(11):1442-1453.

20. Ferkingstad E, Sulem P, Atlason BA, Sveinbjornsson G, Magnusson MI, Styrmisdottir EL, Gunnarsdottir K, Helgason A, Oddsson A, Halldorsson BV *et al*: **Large-scale integration of the plasma proteome with genetics and disease**. *Nat Genet* 2021, **53**(12):1712-1721.

21. Carter AR, Sanderson E, Hammerton G, Richmond RC, Davey Smith G, Heron J, Taylor AE, Davies NM, Howe LD: **Mendelian randomisation for mediation analysis: current methods and challenges for implementation**. *Eur J Epidemiol* 2021, **36**(5):465-478.

22. Zuber V, Grinberg NF, Gill D, Manipur I, Slob EAW, Patel A, Wallace C, Burgess S: **Combining evidence from Mendelian randomization and colocalization: Review and comparison of approaches**. *Am J Hum Genet* 2022, **109**(5):767-782.

23. Giambartolomei C, Vukcevic D, Schadt EE, Franke L, Hingorani AD, Wallace C, Plagnol V: **Bayesian test for colocalisation between pairs of genetic association studies using summary statistics**. *PLoS Genet* 2014, **10**(5):e1004383.
